# Differential Ganglioside and Cholesterol Depletion by Various Cyclodextrin Derivatives and Their Effect on Synaptosomal Glutamate Release

**DOI:** 10.3390/ijms23169460

**Published:** 2022-08-21

**Authors:** Orsolya Geda, Tamás Tábi, Péter P. Lakatos, Éva Szökő

**Affiliations:** Department of Pharmacodynamics, Semmelweis University, 4 Nagyvárad tér, H-1089 Budapest, Hungary

**Keywords:** gangliosides, cyclodextrines, cholesterol, membrane lipids, membrane microdomains, lipid rafts, synaptosomes, glutamate release, synaptic transmission

## Abstract

Gangliosides are glycosphingolipids of the plasma membrane and are highly enriched in the nervous system where they play a vital role in normal cell functions. Furthermore, several studies suggest their potential involvement in the pathogenesis of neurological conditions. Since cyclodextrins (CDs) can form inclusion complexes with various lipids, methylated beta-CDs are widely used in biomedical research to extract cholesterol from the membrane and study its cellular role. Despite CDs being known to interact with other membrane lipid components, their effect on gangliosides is poorly characterized. The aim of this research was to investigate the effect of dimethyl-beta-cyclodextrin (DIMEB), hydroxypropyl-beta-cyclodextrin (HPBCD), randomly methylated-alpha-cyclodextrin (RAMEA), and hydroxypropyl-alpha-cyclodextrin (HPACD) on ganglioside and cholesterol levels in rat brain synaptosomes. Their effect on membrane integrity and viability was also assessed. We examined the role of lipid depletion by CDs on the release of the major excitatory neurotransmitter, glutamate. Selective concentration range for cholesterol depletion was only found with HPBCD, but not with DIMEB. Selective depletion of gangliosides was achieved by both RAMEA and HPACD. The inhibition of stimulated glutamate release upon ganglioside depletion was found, suggesting their potential role in neurotransmission. Our study highlights the importance of the characterization of the lipid depleting capability of different CDs.

## 1. Introduction

Gangliosides are glycosphingolipids (GSLs) of the plasma membrane, and are highly abundant in the nervous system where they are considered to play a key role in several signaling pathways and cell adhesion. Their structures undergo major changes during embryonic development [1] and their distribution showed large differences between brain areas [2]. Early studies also showed that they can induce neurite outgrowth in vitro by their exogenous addition to cells [3,4]. These findings and their abundance in the nervous system compared to extraneural tissues led to the hypothesis that they play a major role in normal neuronal functions. Genetic models in mice, where ganglioside biosynthesis is targeted, showed that gangliosides are important in normal myelin-axon stabilization [5] and their presence was found to be essential for normal brain development [6].

Furthermore, there is growing evidence that their altered levels or composition are possibly involved in neurodegenerative diseases (e.g., Alzheimer’s disease and Parkinson’s disease [7]), however, their exact role in the pathogenesis is not fully understood.

Gangliosides are reported to be enriched in microdomains of the membrane, tightly packed together with cholesterol and sphingomyelin [8]. These membrane microdomains or lipid rafts are considered to create a relatively ordered phase compared to the surrounding membrane that acts as a platform and influences the organization of cell signaling molecules. In the membrane, gangliosides are mainly inserted into the external leaflet with their hydrophobic ceramide moiety constituted by a long-chain amino alcohol, sphingosine connected to a fatty acid by an amide linkage. Their variable oligosaccharide chain is composed of a different number of neutral sugars and at least one sialic acid that provides a negative charge at physiological pH. Both their sugar chain and the lipid tail have specific physicochemical properties that favor the formation of microdomains. For example, their capability of forming a hydrogen-bond network at the lipid–water interface, the geometry of the oligosaccharide headgroup, the carbohydrate–water interactions as well as their long, saturated alkyl chains are all features that strongly suggest a role in the formation of liquid-ordered microdomains in the membrane [8]. Cholesterol is known to modulate membrane fluidity and interact preferentially with long, saturated alkyl chains such as those found in gangliosides, whose interaction also supports the formation of liquid-ordered microdomains [9]. It should be noted that although the lipid raft hypothesis is widely accepted, there is also skepticism about whether rafts exist due to the technical challenges of studying them in living cells [10,11].

Nevertheless, there are numerous reports on the modulating effect of gangliosides on receptor tyrosine kinases such as EGFR, PDGFR, FGFR, and insulin receptor [7], while other studies have suggested that lipid rafts, and specifically gangliosides, may be involved in nociceptive mechanisms via the regulation of the nociceptive cation channel, TRPV1 [12,13]. The role of lipid rafts was also studied in neurotransmitter signaling, and several neurotransmitter receptors (NMDA, AMPA, GABA_A_) and transporters (serotonin, norepinephrine, glutamate transporter) have been shown to localize in membrane microdomains [14]. In neurotransmission, specifically, the role of gangliosides has been studied and their influence on ion transport [15] and exocytotic process revealed.

Cyclodextrins (CDs) are cyclic oligosaccharides extensively used in drug formulation to improve the water solubility and bioavailability of drugs. Alpha-, beta-, and gamma-cyclodextrins (ACDs, BCDs, and GCDs) consist of 6, 7, or 8 α-1,4-glucopyranose units, respectively. CDs have a hydrophilic surface and hydrophobic internal cavity with a different inner diameter, depending on the number of glucopyranose units. They can form inclusion complexes with a broad range of hydrophobic molecules. BCD and its derivatives are the most widely used CDs in medicinal products due to the compatibility of their cavity size with numerous drugs, but native ACD and GCD can also be found in some formulations [16]. Substitution of the hydrogen bond-forming secondary hydroxyl groups results in a significant improvement in the aqueous solubility of BCDs [17]. Hydroxypropyl-beta-cyclodextrin (HPBCD), sulfobutyl ether-beta-cyclodextrin (SBEBCD), or randomly methylated-beta-cyclodextrin (RAMEB) are examples of chemically modified BCDs used as drug excipients [16]. In addition, HPBCD is under clinical investigation as a drug candidate in Niemann–Pick type C (NPC) disease, where it has been reported to alleviate cholesterol accumulation in lysosomes [18]. 

Several studies have shown that CDs interact with membrane components [19,20]. BCDs, especially methylated beta-cyclodextrins (MBCDs), have a high affinity to cholesterol and are used extensively to manipulate the plasma membrane cholesterol level and consequently to disrupt lipid rafts [21]. 

Despite the extensive use of CDs, their effect on gangliosides is poorly characterized [21,22,23]. Our previous capillary electrophoresis (CE) experiments showed that several CDs including dimethyl-beta-cyclodextrin (DIMEB), HPBCD, hydroxypropyl-alpha-cyclodextrin (HPACD,) and randomly methylated-alpha-cyclodextrin (RAMEA) form complexes with gangliosides [24]. CDs most likely interact with the ceramide portion of gangliosides because it is known that BCDs and ACDs are able to form complexes with long chain fatty acids [25].

The aim of this study was to examine the effect of various CDs on gangliosides and the cholesterol content of rat brain synaptosomes as well as on the viability and membrane integrity. The following CDs, having a different cavity size and substituents were tested: DIMEB, HPBCD, RAMEA, and HPACD. The influence of membrane modification by CDs on synaptic glutamate release was also assessed.

## 2. Results and Discussion

### 2.1. Effect of CDs on Ganglioside and Cholesterol Content of Synaptosomes

Ganglioside and cholesterol depleting effects of the CDs were examined by incubating rat cerebral synaptosomes in the presence of their various concentrations, followed by the analysis of the ganglioside and cholesterol content that remained in the synaptosomes. 

**Figure 1:**
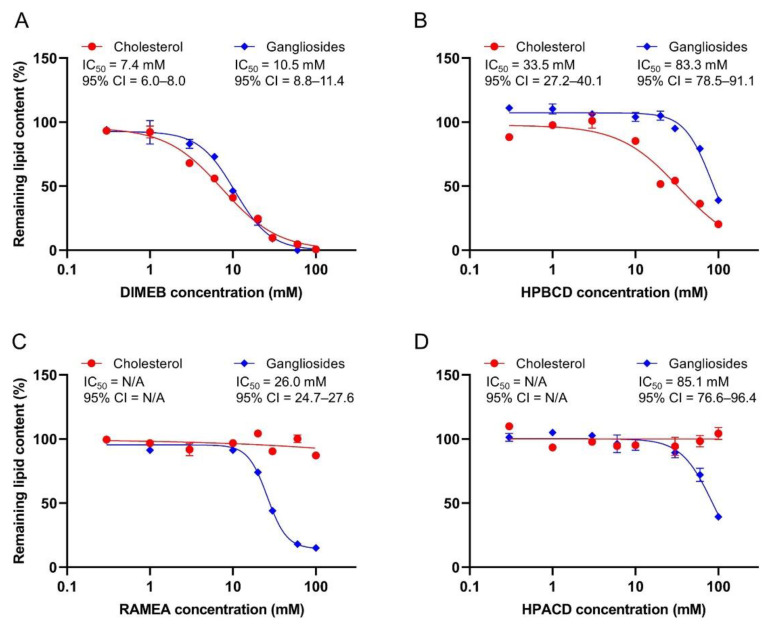
The effect of DIMEB (**A**), HPBCD (**B**), RAMEA (**C**), and HPACD (**D**) on the ganglioside and cholesterol content of the rat cerebral synaptosomes. Lipid levels were measured after 40 min of exposure of the synaptosomes to cyclodextrins. Values are expressed as the percentage of vehicle-treated synaptosomes. The IC_50_ values and their 95% confidence intervals (95% CI) are given. Data are represented as the means ± SEM (n = 3). N/A means non applicable.

We found that DIMEB potently extracted both the cholesterol and gangliosides from the synaptosomes. IC_50_ values of 7.4 mM and 10.5 mM were estimated for the cholesterol and ganglioside depletion, respectively, and no selective concentration range for cholesterol depletion was identified. Nearly total extraction of both lipids was found at 30 mM DIMEB concentration (Figure 1A).

We found that another extensively used BCD derivative, HPBCD, also depleted both cholesterol (IC_50_ = 33.5 mM) and gangliosides (IC_50_ = 83.3 mM), but less potently than DIMEB did. A significant difference in its cholesterol and ganglioside extracting ability was detected and negligible ganglioside loss (~5%) was seen at 50% cholesterol depleting concentration (Figure 1B). 

According to our present results, ACD derivatives, having a smaller cavity size, were not able to deplete cholesterol from synaptosomes in the tested concentration range (Figure 1C,D). In contrast, both RAMEA and HPACD depleted the gangliosides, where RAMEA was found to be more potent (IC_50_ = 26.0 mM) than HPACD (IC_50_ = 85.1 mM). The latter caused only ~60% extraction, even at the highest examined CD concentration (100 mM).

In the majority of experiments, methylated BCDs are used as cholesterol depleting agents, but their selectivity over other lipids has not been fully characterized. Ottico and co-workers [26] reported that cerebellar granule cells treated with MBCD resulted in a reduction in the sphingomyelin and GSL levels in addition to the cholesterol extraction. In line with these results, our findings confirm a considerable ganglioside depleting capability of DIMEB. The biological effects of MBCDs thus cannot be solely explained by cholesterol removal since they can also interact with other membrane components. 

The inferior potency of HPBCD compared to methylated BCDs in cholesterol depletion was previously reported by other authors [19,26], which is the likely reason of its less common use in in vitro experiments. Its effects on other membrane constituents have hardly been examined, however, a study on its potential mechanism in NPC revealed that it reduces the accumulation of GM2 and GM3 immunoreactivity in a mouse model [22]. Based on our results, HPBCD is not entirely selective for cholesterol, but in an appropriate concentration range, selective depletion can be achieved. 

In our studies, cholesterol depletion was not observed upon treatment with ACD derivatives, which is in line with our expectations based on the data in the literature [20,27,28]. The found ganglioside depleting capability of both RAMEA and HPACD is in accordance with our previous CE experiments, where the complex formation of gangliosides with ACD derivatives was detected [24]. Among the ACDs, mainly the interactions of native ACD with membrane components have been previously studied and the depletion of phospholipids was reported [20,28]. In terms of the ACD derivatives, two studies reported a change in the ganglioside immunoreactivity. Maeda and co-workers [23] found that both DIMEA (hexakis(2,6-di-O-methyl)-alpha-cyclodextrin) and HPACD reduced lysosomal accumulation of GM1 in a cell model of GM1 gangliosidosis by immunofluorescence staining. The methylated derivative of ACD was more effective, similar to our present results. Davidson and co-workers [22] studied HPACD and found it to be much less effective compared to HPBCD in reducing the immunoreactivity accumulation of the GM2 and GM3 gangliosides.

In our present work, the concentration–response curves for the depletion of each neuronal ganglioside were also constructed individually and their IC_50_ values were estimated (Table 1). No reasonable difference in the individual IC_50_ values was found in the case of any tested CD derivatives, therefore, the concentration dependence of the total ganglioside depletion is shown in Figure 1.

### 2.2. Effect of CDs on Membrane Integrity and Viability of Synaptosomes

As CD treatments induced considerable changes in the membrane lipid composition, we aimed at assessing whether they interfered with the membrane integrity and viability of the synaptosomes. The release of the cytosolic LDH enzyme was used as an indicator of compromised membrane integrity and a reduction in resazurin by metabolically active synaptosomes was applied as a measure of viability.

**Figure 2:**
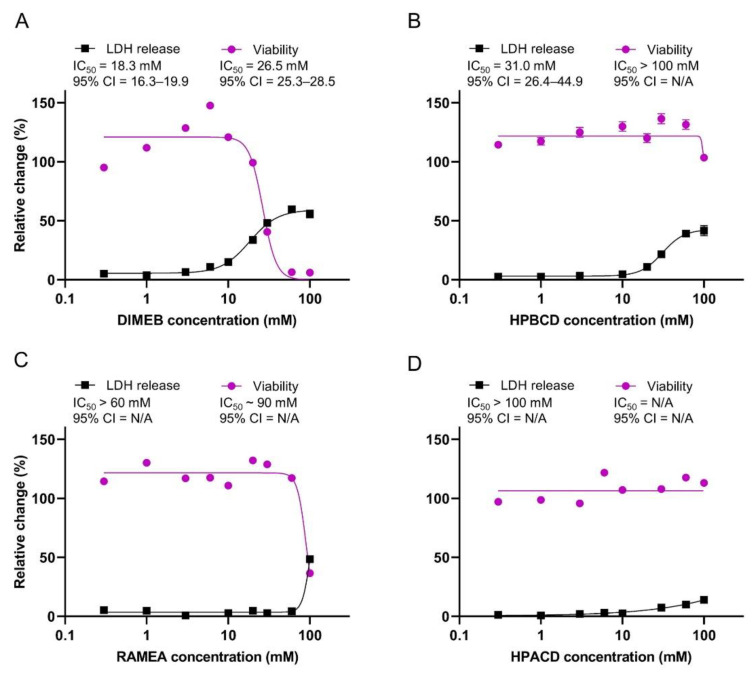
The effect of DIMEB (**A**), HPBCD (**B**), RAMEA (**C**), and HPACD (**D**) on the membrane integrity and viability of the rat cerebral synaptosomes. Synaptosomes were analyzed after 40 min exposure to cyclodextrins. For estimating membrane integrity, LDH release from synaptosomes was calculated as percentage of total LDH content determined by Triton X-100 lysis. Viability was measured by resazurin reduction assay and values are expressed as the percentage of vehicle-treated synaptosomes. IC_50_ values and their 95% confidence intervals (95% CI) are given. Data are represented as means ± SEM (n = 3). N/A means non applicable.

DIMEB treatment resulted in a concentration dependent increase in the LDH release with an IC_50_ value of 18.3 mM. Interestingly, the effect reached a plateau at about 60% LDH release (Figure 2A). At the same time, DIMEB caused a significant decrease in the metabolic activity of synaptosomes in concentrations higher than 10 mM. The IC_50_ value was 26.5 mM and the total loss of viability was observed at 60 mM DIMEB concentration (Figure 2A).

In accordance with its lower cholesterol removal potency, HPBCD induced membrane damage at higher concentrations (IC_50_ = 31.0 mM) than DIMEB did (Figure 2B) and similarly had a maximal effect of a 43% LDH release. A significant decrease in the viability was observed only at the highest examined, 100 mM HPBCD concentration (Figure 2B).

The tested ACD derivatives induced less damage in the synaptosomes than the BCD derivatives did. RAMEA had an influence on the membrane integrity and viability only at the highest tested 100 mM concentration (Figure 2C), while HPACD had no effect on the viability and only a mild increase in the LDH release was seen even at this high concentration (Figure 2D).

Our results on the synaptosomes are in line with previous findings obtained on cells, suggesting that cholesterol depletion plays a major role in membrane damage and reduced viability. Correlation between cholesterol complexation and cytotoxicity as well as with hemolytic activity [29,30] was previously reported, supporting the hypothesis that the toxic effect of CDs highly depends on their cholesterol extraction capacity from the plasma membrane as cholesterol is the main rigidifier in plasma membranes. However, ACDs that are unable to complex cholesterol, also had some damaging activity that might be explained by their interaction with other membrane components (e.g., phospholipids [20]) in high concentration. 

Our results are in line with previous findings that among both the ACDs and BCDs, methylated derivatives were the most toxic. Methylated BCDs were reported to induce hemolysis in lower concentrations than other BCD derivatives [29,31] and a similar tendency was shown in their cytotoxic effect by many authors using various cell lines [29,32,33,34]. The toxicity of the ACD derivatives was recently compared by Róka and co-workers [35] and methylated derivatives were the most toxic. 

According to our results, the LDH release assay, which is commonly used to assess cell death, showed enhanced membrane permeability rather than direct toxicity in the case of the CD treatments since lipid depletion, even at 100 mM CD concentration, did not induce the total loss of membrane integrity. The complete reduction in viability (measured by resazurin test) found in the case of DIMEB treatment, however, can probably be explained by the massive leakage of small molecules that are essential for normal metabolic activity. In accordance, Ottico and co-workers [26] reported that MBCD treatment increased the membrane permeability, which was partially reversed by cholesterol repletion. These findings indicate that the LDH release assay in the case of CD treatments, where alterations are induced in membrane components, is not a true estimate of cytotoxicity, therefore, a complementary assay assessing the metabolic activity is also required.

### 2.3. Effect of CDs on Basal and 4-Aminopyridine (4-AP)-Evoked Glutamate Release from Synaptosomes

While there are several articles on the involvement of membrane cholesterol in the regulation of neurotransmitter release from synapses [36,37], the possible role of gangliosides has been mainly unexplored thus far. Therefore, we investigated the influence of the four CDs on glutamate release from rat cerebral synaptosomes to reveal whether lipid depletion had an effect on synaptic activity. To selectively deplete gangliosides, RAMEA and HPACD were used in their 50% ganglioside extracting concentrations (26 mM and 85 mM, respectively). Selective cholesterol depletion was obtained by the application of 33 mM HPBCD corresponding to 50% cholesterol and negligible ganglioside extraction. The treatment of synaptosomes with 10 mM DIMEB concentration induced similar, approximately 50% ganglioside and cholesterol extraction. At these concentrations, CDs had no effect on the viability and only minor effect on the membrane integrity. Basal (non-stimulated) and 4-AP-evoked glutamate release were measured from the CD-treated synaptosomes.

**Figure 3:**
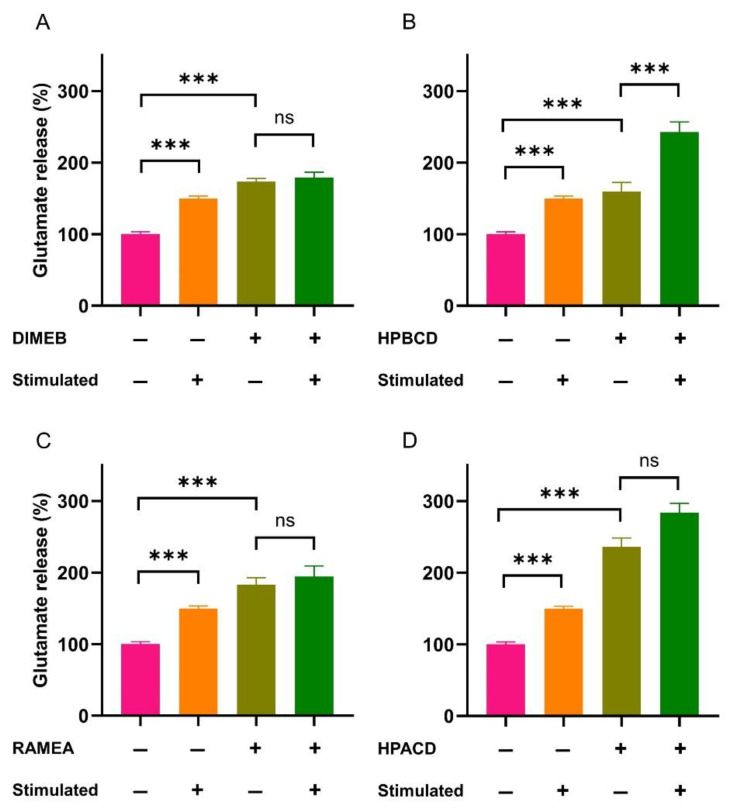
The basal (non-stimulated) and 4-aminopyridine-evoked release of glutamate from rat cerebral synaptosomes upon DIMEB (**A**), HPBCD (**B**), RAMEA (**C**), and HPACD (**D**) treatment. Glutamate release was measured after 40 min of exposure of the synaptosomes to cyclodextrins. Glutamate release is expressed as the percentage of baseline release from the vehicle-treated synaptosomes. Data are represented as the means ± SEM (n = 8). One-way ANOVA followed by Bonferroni’s *post hoc* test was used for data evaluation. *** *p* ≤ 0.001, ns: not significant.

All four CDs significantly increased the basal glutamate release from the synaptosomes (Figure 3). Furthermore, the stimulated glutamate release was not detected in the RAMEA, HPACD, and DIMEB-treated groups, where significant ganglioside depletion was induced. In the case of HPBCD that selectively depleted cholesterol, 4-AP-stimulation still evoked glutamate release, similar to the vehicle-treated synaptosomes.

The increased basal release of glutamate may be the consequence of mildly compromised membrane integrity, resulting in a steady leakage of the neurotransmitter. Our results also suggest that stimulated neurotransmitter release is more dependent on ganglioside, rather than the cholesterol content of the synaptic membranes. 

Previously, it has shown that several proteins involved in exocytosis are localized in membrane microdomains such as syntaxin and SNAP-25 [38,39], voltage-gated Ca^2+^, and K^+^ channels [40,41,42]. Localization of these proteins was examined mainly by disrupting the membrane microdomains with MBCD based on its well-known cholesterol depleting capacity. It was also reported that MBCD treatment impairs the exocytotic release of glutamate from rat brain synaptosomes [43,44] and that of dopamine from PC12 cells, in line with our present results. In these studies, the effect of the MBCD treatments was mostly interpreted as a result of cholesterol depletion. However, our results showed that DIMEB depletes gangliosides to a similar extent compared to cholesterol, therefore, it can be assumed that the reported effects of MBCD treatments cannot be attributed solely to cholesterol extraction. Furthermore, the involvement of gangliosides in neurotransmitter release was examined by their direct modulation. Exogenous administration of GM1 and GQ1b was reported to enhance the depolarization-induced Ca^2+^ influx and release of acetylcholine from rat brain synaptosomes [45]. The inhibition of ganglioside synthesis was also shown to reduce glutamate release from the neuro2a neuroblastoma cell line [46], confirming the role of gangliosides in neurotransmitter release. In accordance with these reports, we found decreased depolarization-evoked glutamate release only when ganglioside depletion was induced, but not upon selective cholesterol extraction. 

## 3. Materials and Methods

### 3.1. Chemicals

All of the reagents used in the experiments were of analytical grade. Boric acid, HEPES, sucrose, glucose, L-glutamate, L-cysteic acid, dimethyl sulfoxide (DMSO), octyl-β-D-thioglycopyranoside (internal standard for ganglioside analysis), sodium hydroxide (NaOH), 4-aminopyridine (4-AP), and other buffer components were obtained from Merck (Darmstadt, Germany). Fetal bovine serum (FBS) was purchased from Biosera (Nuaille, France). Methanol and chloroform used for liquid–liquid extraction were obtained from Molar Chemicals Ltd. (Budapest, Hungary). Ganglioside standards (GM1, GD1a, GD1b, GT1b, and GQ1b) used for ganglioside analysis were products of the Cayman Chemical Company (Ann Arbor, MI, USA). Beta-cyclodextrin (BCD), heptakis(2,6-di-O-methyl)-beta-cyclodextrin (DIMEB) with isomeric purity >35% DS~14, (2-hydroxypropyl)-beta-cyclodextrin (HPBCD) DS~4.5, randomly methylated alpha-cyclodextrin (RAMEA) DS~11 and (2-hydroxypropyl)-alpha-cyclodextrin (HPACD) DS~4.5 were purchased from Cyclolab Ltd. (Budapest, Hungary). 4-Fluoro-7-nitrobenzofurazan (NBD-F) was obtained from the Tokyo Chemical Industry (Tokyo, Japan). DL-Threo-β-benzyloxyaspartic acid (DL-TBOA) was a product of Tocris Bioscience (Bristol, United Kingdom). Ultrapure water was obtained from a Milli-Q Direct 8 System (Merck, Darmstadt, Germany).

### 3.2. Preparation and Treatment of Synaptosomes with Cyclodextrins

Adult male Wistar rats purchased from Toxi-Coop Ltd. (Budapest, Hungary) were used in the experiments. Cerebral synaptosomes were prepared according to Modi and co-workers [47] with the following modifications. Animals were decapitated and the brain was removed rapidly. Isotonic sucrose buffer (0.32 M sucrose, 4 mM HEPES-NaOH, pH 7.4) was used to homogenize the cerebrum with a Potter S Homogenizer (B. Braun, Melsungen, Germany) with 15 strokes at 1000 rpm. The homogenate was centrifuged (1500× *g*, 10 min, 4 °C) and the pellet was resuspended in sucrose buffer and centrifuged with the same parameters again. The two supernatants were combined and centrifuged (20,000× *g*, 10 min, 4 °C) to pellet the crude synaptosomes. The resulting supernatant was discarded and the pellet was washed again with an equal volume of sucrose buffer and then pelleted by centrifugation (20,000× *g*, 10 min, 4 °C). Synaptosomes were stored frozen using cryopreservation buffer and the slow freezing method [48,49] to preserve their physiological state and integrity. Briefly, synaptosome pellets were resuspended in isotonic sucrose buffer containing 10% DMSO (*v*/*v*) and 10% FBS (*v*/*v*). To slow down the freezing process at −80 °C, synaptosomes were placed in a 3 cm thick-walled polystyrene box with a lid. Synaptosomes were stored at −80 °C until use. Immediately prior to the experiments, synaptosomes were rapidly thawed in a 37 °C water bath and pelleted by centrifugation (20,000× *g*, 10 min, 4 °C) to remove the cryopreservation buffer. The pellet was resuspended in isotonic HEPES-glucose buffer (10 mM HEPES-NaOH, 130 mM NaCl, 5.4 mM KCl, 1.3 mM CaCl_2_, 0.9 mM MgCl_2_, 5.5 mM glucose, pH 7.4) and aliquoted according to the experimental design.

For all types of experiments, except for the glutamate release measurements, vehicle-treated (control) and CD-treated synaptosomes were incubated in 500 μL HEPES-glucose buffer with or without CDs, respectively, for 40 min at 37 °C. In these experiments, the CD solutions contained DIMEB, HPBCD, RAMEA, or HPACD in various concentrations (0.3–100 mM). After incubation, the synaptosomes were centrifuged (20,000× *g*, 5 min, 4 °C) and used immediately for further analysis. A slightly different incubation method used in the glutamate release measurements is described in the corresponding section. Across all types of experiments, osmolarities of the CD solutions were adjusted and they were between 290 and 310 mOsm. At least three parallel measurements were carried out.

### 3.3. Ganglioside Analysis

The major neuronal ganglioside (GM1, GD1a, GD1b, GT1b, GQ1b) content of the rat cerebral synaptosome samples were measured by our previously published validated method [24]. Briefly, after the CD treatments, gangliosides were extracted from 10 mg synaptosomes by liquid–liquid extraction. Ganglioside extracts were evaporated to dryness under a gentle stream of nitrogen (Zymark TurboVap LV, Hopkinton, MA, USA) in a 37 °C water bath. The dry ganglioside extract yielded from 10 mg synaptosomes was resuspended in 60 µL water, and centrifuged (20,000× *g*, 5 min, 4 °C) to remove the precipitate and the supernatant was analyzed by CE.

Ganglioside analysis was performed on a P/ACE MDQ capillary electrophoresis system (Beckman Coulter Inc., Brea, CA, USA) with UV detection at 200 nm. Uncoated fused silica capillaries with 75 µm internal diameter, 70 and 80 cm total and effective capillary length, respectively (Agilent Technologies, Santa Clara, CA, USA), were used. Separation was achieved by applying +25 kV voltage in a separation buffer containing 20 mM RAMEA and 100 mM sodium borate at pH 10.0. Ganglioside depletion in the CD-treated synaptosomes was calculated as the percentage of the ganglioside content of the vehicle-treated synaptosomes.

### 3.4. Cholesterol Assay

The cholesterol levels of the rat cerebral synaptosomes were measured with a Cholesterol Fluorometric Assay Kit (Cayman Chemical Company, Ann Arbor, MI, USA) with minor adjustments for the sample preparation. Briefly, after CD treatments, 2 mg of the synaptosomes were extracted with 10 vol chloroform–methanol 2:1 (*v*/*v*) to gain a lipid extract. The samples were centrifuged (20,000× *g*, 5 min, 4 °C) and then the supernatant was evaporated to dryness under a nitrogen stream (Zymark TurboVap LV) in a 37 °C water bath. The extracts were resuspended and diluted in the assay buffer (1.2 mL/mg synaptosome). Further preparation of the assay was according to the manufacturer’s protocol. Fluorescence was measured with a Varioskan LUX multimode microplate reader (Thermo Fisher Scientific, Waltham, MA, USA) at 530/590 nm. Cholesterol depletion in the CD-treated synaptosomes was calculated as the percentage of the cholesterol content of the vehicle-treated synaptosomes.

### 3.5. Resazurin Reduction Viability Assay

Viability of the rat cerebral synaptosomes was measured by the Resazurin Cell Viability Kit (Cell Signaling Technology Inc., Danvers, MA, USA) with minor modifications. The assay was based on the reduction in the nonfluorescent resazurin to highly fluorescent resorufin by intracellular dehydrogenase enzymes. Briefly, after CD treatments, 2 mg of the synaptosomes were centrifuged (20,000× *g*, 5 min, 4 °C) and the supernatant was discarded. The synaptosome pellet was then gently resuspended in HEPES-glucose buffer (pH 7.4) containing 10% resazurin solution. After 25 min of incubation, synaptosomes were centrifuged (20,000× *g*, 5 min, 4 °C). Fluorescence of the supernatant was measured with a Varioskan LUX multimode microplate reader (Thermo Fisher Scientific) at 530/590 nm. Viability of the CD-treated synaptosomes was calculated as the percentage of vehicle-treated control synaptosomes.

### 3.6. Membrane Integrity Measurement by LDH Release Assay

The membrane integrity of the synaptosomes was measured by the CytoTox-ONE Homogeneous Membrane Integrity Assay (Promega, Madison, WI, USA) with minor modifications. The assay measures the release of the lactate dehydrogenase (LDH) enzyme from synaptosomes with damaged membranes. 

Briefly, after the CD treatments, 2 mg of the synaptosomes was centrifuged (20,000× *g*, 5 min, 4 °C) and the supernatant was collected to measure the LDH release from the synaptosomes. The synaptosome pellet was lysed with 1% Triton X-100 solution (*v*/*v*) to determine the total LDH content. Both the supernatant and lysis samples were diluted 10-fold. The LDH activity was then assessed according to the manufacturer’s instructions and the fluorescence was measured by a Varioskan LUX multimode microplate reader (Thermo Fisher Scientific) at 530/590 nm. The LDH release from the synaptosomes was calculated as the percentage of the total LDH content determined by Triton X-100 lysis.

### 3.7. Glutamate Release Measurement

After thawing and centrifugation, the cryopreserved synaptosomes were resuspended in isotonic HEPES-glucose buffer. Synaptosomal suspensions corresponding to 10 mg of synaptosomes were centrifuged to an 8-well strip plate (15 min, 2500× *g*, 4 °C) and the supernatant was discarded. Vehicle-treated (control) and CD-treated synaptosomes were incubated in 200 μL HEPES-glucose buffer with or without CDs, respectively, for 40 min at 37 °C. The CD solutions contained 10 mM DIMEB, 34 mM HPBCD, 26 mM RAMEA, or 85 mM HPACD. After incubation, the supernatant was replaced with fresh HEPES-glucose buffer containing 40 µM DL-TBOA (a competitive, non-transportable blocker of excitatory amino acid transporters that inhibits the reuptake of released glutamate [50]) and equilibrated for 2 × 10 min at 37 °C with buffer replacement. After equilibration, the buffer was replaced by fresh HEPES-glucose buffer in the case of unstimulated groups. To evoke depolarization, the buffer was replaced by HEPES-glucose buffer containing 1 mM 4-AP. Following stimulation, aliquots were taken from the buffer at 8 min and stored at −20 °C until CE analysis. 

The glutamate content of the aliquots was analyzed by a capillary electrophoresis-laser induced fluorescence method developed in our laboratory [51]. Briefly, samples were subjected to derivatization with NBD-F (1 mg/mL final concentration) in 20 mM borate buffer, pH 8.5 for 20 min at 65 °C. A total of 1 µM L-cysteic acid was used as the internal standard. The derivatized samples were analyzed by a P/ACE MDQ Plus capillary electrophoresis system (SCIEX, Framingham, MA, USA) equipped with a laser source of excitation and emission wavelengths of 488 and 520 nm, respectively. Separation was carried out in polyacrylamide-coated fused silica capillaries with a 75 µm internal diameter, and a 10 and 50 cm effective and total capillary length, respectively (Agilent Technologies). The separation buffer contained 8 mM BCD and 100 mM sodium borate at pH 8.5. Glutamate release was normalized to the baseline release from the vehicle-treated, unstimulated synaptosomes.

### 3.8. Statistical Analysis

Data were analyzed by GraphPad Prism 8 (GraphPad Software, La Jolla, CA, USA). Concentration–response curves were constructed by the nonlinear regression method. One-way ANOVA followed by Bonferroni’s post hoc test was used for the data analysis in the case of the glutamate release experiments. All data are presented as means ± SEM of at least three parallel measurements. Differences were considered significant if *p* < 0.05.

## 4. Conclusions

Our present results demonstrated that the CD derivatives tested could effectively extract gangliosides from the rat brain synaptosomes. Since gangliosides are involved in many cellular functions, the ganglioside-removing ability of these CDs should be taken into consideration in their application. Methylated BCDs are commonly used as cholesterol-depleting agents, however, we showed that DIMEB extracts both cholesterol and gangliosides to a similar extent. Our present results suggest that HPBCD is more suitable for selective cholesterol depletion than DIMEB due to the significant difference between its cholesterol and ganglioside extracting concentration, and its lower toxicity is also favorable. Both RAMEA and HPACD were able to extract gangliosides without affecting the cholesterol content, therefore, they may be appropriate for the selective depletion of gangliosides. We also showed that ganglioside, but not cholesterol depletion, interfered with stimulated glutamate release from the rat brain synaptosomes.

Our results thus emphasize the significance of gangliosides in various membrane functions. Moreover, the importance of the characterization of the lipid depleting capability of different CDs also highlighted that it is a prerequisite for the examination of the specific role of individual lipid classes.

## Figures and Tables

**Table 1:** The IC_50_ values and their 95% confidence intervals (95% CI) of the depletion of individual and total ganglioside content upon DIMEB, HPBCD, RAMEA, and HPACD treatment of the rat cerebral synaptosomes.

	DIMEB		HPBCD		RAMEA		HPACD	
	IC_50_ (mM)	95% CI	IC_50_ (mM)	95% CI	IC_50_ (mM)	95% CI	IC_50_ (mM)	95% CI
GM1	15.0	12.7–17.6	80.8	76.0–89.5	29.1	27.9–30.5	86.7	79.0–100.7
GD1a	9.7	7.3–15.0	83.7	78.6–90.4	25.1	23.4–27.2	72.9	65.6–82.7
GD1b	14.5	10.7–23.9	86.6	80.2–98.0	26.4	24.6–28.3	90.6	68.2–139.9
GT1b	8.7	7.9–9.6	80.1	74.6–90.5	24.2	22.8–25.6	104.8	93.9–133.5
GQ1b	11.4	9.8–13.4	85.1	71.3–105.0	30.3	26.3–42.0	61.2	45.5–72.2
Total	10.5	8.8–11.4	83.3	78.5–91.1	26.0	24.7–27.6	85.1	76.6–96.4

The depletion of individual neuronal gangliosides (GM1, GD1a, GD1b, GT1b, GQ1b) is shown. The total ganglioside levels are given as the sum of individual ganglioside ones. The values were estimated by concentration–response nonlinear fitting.

## Data Availability

The data that support the findings of this study are available from the corresponding author upon reasonable request.
